# HIVBrainSeqDB: a database of annotated HIV envelope sequences from brain and other anatomical sites

**DOI:** 10.1186/1742-6405-7-43

**Published:** 2010-12-14

**Authors:** Alexander G Holman, Megan E Mefford, Niall O'Connor, Dana Gabuzda

**Affiliations:** 1 Department of Cancer Immunology and AIDS, Dana-Farber Cancer Institute, Dana-Farber Cancer Institute, 44 Binney Street, Boston, Massachusetts, 02115, USA; 2 Center for Cancer Computational Biology, Dana-Farber Cancer Institute, Dana-Farber Cancer Institute, 44 Binney Street, Boston, Massachusetts, 02115, USA; 3 Department of Neurology, Harvard Medical School, 25 Shattuck Street, Boston, Massachusetts, 02115, USA

## Abstract

**Background:**

The population of HIV replicating within a host consists of independently evolving and interacting sub-populations that can be genetically distinct within anatomical compartments. HIV replicating within the brain causes neurocognitive disorders in up to 20-30% of infected individuals and is a viral sanctuary site for the development of drug resistance. The primary determinant of HIV neurotropism is macrophage tropism, which is primarily determined by the viral envelope (*env*) gene. However, studies of genetic aspects of HIV replicating in the brain are hindered because existing repositories of HIV sequences are not focused on neurotropic virus nor annotated with neurocognitive and neuropathological status. To address this need, we constructed the HIV Brain Sequence Database.

**Results:**

The HIV Brain Sequence Database is a public database of HIV envelope sequences, directly sequenced from brain and other tissues from the same patients. Sequences are annotated with clinical data including viral load, CD4 count, antiretroviral status, neurocognitive impairment, and neuropathological diagnosis, all curated from the original publication. Tissue source is coded using an anatomical ontology, the Foundational Model of Anatomy, to capture the maximum level of detail available, while maintaining ontological relationships between tissues and their subparts. 44 tissue types are represented within the database, grouped into 4 categories: (i) brain, brainstem, and spinal cord; (ii) meninges, choroid plexus, and CSF; (iii) blood and lymphoid; and (iv) other (bone marrow, colon, lung, liver, etc). Patient coding is correlated across studies, allowing sequences from the same patient to be grouped to increase statistical power. Using Cytoscape, we visualized relationships between studies, patients and sequences, illustrating interconnections between studies and the varying depth of sequencing, patient number, and tissue representation across studies. Currently, the database contains 2517 envelope sequences from 90 patients, obtained from 22 published studies. 1272 sequences are from brain; the remaining 1245 are from blood, lymph node, spleen, bone marrow, colon, lung and other non-brain tissues. The database interface utilizes a faceted interface, allowing real-time combination of multiple search parameters to assemble a meta-dataset, which can be downloaded for further analysis.

**Conclusions:**

This online resource, which is publicly available at http://www.HIVBrainSeqDB.org, will greatly facilitate analysis of the genetic aspects of HIV macrophage tropism, HIV compartmentalization and evolution within the brain and other tissue reservoirs, and the relationship of these findings to HIV-associated neurological disorders and other clinical consequences of HIV infection.

## Introduction

The population of HIV replicating within a host consists of independently evolving and interacting sub-populations, as demonstrated by the various degrees of phylogenetic compartmentalization seen across and within anatomical compartments and various rates of decay in viral load during HAART therapy [1,2]. Several factors contribute to this genetic compartmentalization: (i) viral target cell tropism--HIV infects CD4+ T cells and macrophages in the periphery, and primarily infects macrophages and microglia (and rarely, astrocytes) in the brain [3]; (ii) viral adaptation in response to immune selection pressures that differ between anatomical compartments [3,4]; (iii) physical barriers such as the blood-brain barrier [5]; and (iv) variable antiretroviral drug penetration into different tissues [6,7]. An important viral sub-population is HIV replicating within the brain [8-10]. HIV replicating in the brain causes neurocognitive and neuropathological disorders in up to 20-30% of infected individuals, particularly in later stages of disease; in the era of HAART, HIV-associated neurocognitive disorders (HAND) have emerged as a significant cause of mortality and morbidity [4,6]. Additionally, the brain is a sanctuary site for the development of drug resistance, because poor antiretroviral drug penetration into the CNS leads to sub-therapeutic drug concentrations and incomplete suppression of viral replication [6]. The primary determinant of HIV neurotropism is macrophage tropism, which is primarily determined by genetic variation in the viral envelope (*env*) gene [8]. Phylogenetically related populations of macrophage-tropic virus are found across brain and other macrophage-rich tissues, such as lung and bone marrow [11,12]. Thus, studies of the genetics of HIV replicating in the brain are pertinent to important clinical aspects of HIV, as well as the biology of the virus replicating within specific anatomical compartments.

There are several excellent existing repositories of HIV sequences in the public domain, two of the most widely used being Genbank at the NCBI [13] and the HIV Sequence Database at the Los Alamos National Laboratory (LANL) (http://hiv.lanl.gov). However, neither is focused on neurotropic virus nor contains clinical annotations of neurocognitive and neuropathological diagnosis. Though more than 20 publications have clonally sequenced HIV *env *from the brain, assembling a meta-dataset of these sequences presents significant technical challenges. To address these challenges, we constructed the HIV Brain Sequence Database (HBSD), the first comprehensive database of HIV envelope sequences clonally sequenced from brain and non-brain tissues, which is publicly available at http://HIVBrainSeqDB.org

### The HIV Brain Sequence Database

**Figure 1:**
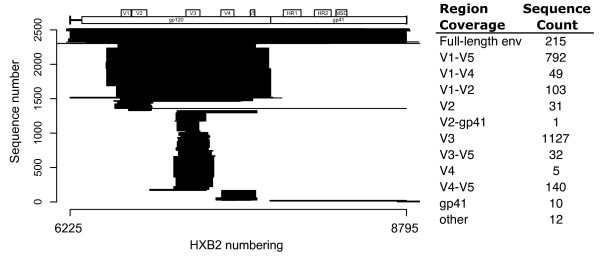
**Sequence coverage of the HIV *env *gene, numbered according to HXB2**. Start and end coordinates are represented, but sequences are not internally aligned so gaps are not represented. The x-axis shows HXB2 nucleotide numbering with a schematic of the *env *gene plotted above. The y-axis shows arbitrary numbering of the plotted sequences.

The HBSD contains 2517 envelope sequences from 90 patients. Sequences were obtained from 22 published studies (Table 1) ranging in publication date from 1991 to 2009 and in number of sequences per publication from 1 to over 700. 1272 of these sequences are brain-derived; the remaining approximately 1245 are derived from blood, lymph node, spleen, bone marrow, colon, lung and other non-brain tissues. 44 independent tissue types are represented within the database. These tissue types are grouped into 4 categories: (i) brain, brainstem, and spinal cord; (ii) meninges, choroid plexus, and CSF; (iii) blood and lymphoid; and (iv) other (bone marrow, lung, liver, etc) (Table 2). Figure 1 shows the database sequence content aligned to the *env *gene of HXB2. V3 region and near full-length gp120 region sequences comprise the majority of the database, with approximately 1100 and 800 sequences, respectively. There are also approximately 200 near full-length *env *sequences, 150 V4-V5 region, and 100 V1-V2 region. As new publications emerge, facilitated by new sequencing technologies, we expect the size of the HBSD to follow the exponential expansion seen by other sequence databases [13].

**Table 1:** Publications describing the cloning of sequences included in the HBSD

Publication	Number of Sequences
Keele, Burton (2008) [19]	51
Power, Chesebro (1994) [20]	15
Peters, Clapham (2004) [21]	31
Mefford, Gabuzda (unpublished)	33
Mefford, Gabuzda (2008) [22]	10
Ohagen, Gabuzda (2003) [23]	35
Thomas, Gabuzda (2007) [24]	55
Liu, Gartner (2000) [25]	31
Martín-García, González-Scarano (2006) [26]	12
Shapshak, Goodkin (1999) [15]	65
Li, Hahn (1991) [27]	2
Gatanaga, Iwamoto (1999) [28]	17
Lamers, McGrath (2009) [29]	715
Salemi, McGrath (2005) [12]	88
Shah, Saksena (2006) [30]	30
Smit, Saksena (2001) [31]	11
Hughes, Simmonds (1997) [32]	87
McCrossan, Simmonds (2006) [18]	259
Morris, Simmonds (1999) [33]	252
Wang, Simmonds (2001) [11]	470
Monken, Srinivasan (1995) [34]	39
Korber, Wolinsky (1994) [17]	209

First author, last author and publication year of included publications, sorted by last author is shown in the left column. Total number of sequences included in the database from each publication is shown in the right column. In some cases, publications may contain additional sequences that did not meet our inclusion criteria--for example, sequences from isolates or patients with no brain sequences--and were therefore omitted.

**Table 2:** Classification of tissues represented in the database, with their respective Foundational Model of Anatomy (FMA) codes

Brain, brainstem, and spinal cord (n = 1272)	FMA Code	Number of sequences
Brain	FMA:50801	171
Brainstem	FMA:79876	16
Caudate nucleus	FMA:61833	7
Cortex of frontal lobe	FMA:242199	67
Cortex of occipital lobe	FMA:242205	20
Cortex of temporal lobe	FMA:242201	77
Frontal lobe	FMA:61824	91
Left frontal lobe	FMA:72970	214
Left hemisphere of cerebellum	FMA:83877	1
Left occipital lobe	FMA:72976	12
Left parietal lobe	FMA:72974	5
Left temporal lobe	FMA:72972	17
Middle frontal gyrus	FMA:61859	10
Occipital lobe	FMA:67325	25
Parietal lobe	FMA:61826	3
Putamen	FMA:61834	1
Right frontal lobe	FMA:72969	43
Right hemisphere of cerebellum	FMA:83876	1
Right occipital lobe	FMA:72975	16
Right parietal lobe	FMA:72973	18
Right temporal lobe	FMA:72971	15
Set of basal ganglia	FMA:84013	87
Spinal cord	FMA:7647	12
Temporal lobe	FMA:61825	41
White matter of frontal lobe	FMA:256178	111
White matter of neuraxis	FMA:83929	29
White matter of occipital lobe	FMA:256188	140
White matter of temporal lobe	FMA:256186	22

**Meninges, choroid plexus, and CSF (n = 184)**		

Choroid plexus of cerebral hemisphere	FMA:61934	44
CSF	FMA:20935	1
Set of meninges	FMA:76821	139

**Blood and lymphoid (n = 776)**		

Blood	FMA:9670	122
Infraclavicular lymph node	FMA:14193	4
Lymph node	FMA:5034	417
Mesenteric lymph node	FMA:12795	28
Peripheral blood mononuclear cell	FMA:86713	15
Spleen	FMA:7196	129
T-lymphocyte	FMA:62870	61

**Other (n = 285)**		

Bone marrow	FMA:9608	31
Colon	FMA:14543	135
Epithelial lining fluid	FMA:276456	5
Liver	FMA:7197	34
Lung	FMA:7195	78
Right lung	FMA:7309	2

### Collection and assembly of HIV sequences

The HBSD attempts to contain all available, published HIV sequences meeting stringent inclusion criteria. For inclusion in the HBSD, sequences must meet the following criteria: (i) be deposited in Genbank; (ii) include some portion of the HIV *env *region; (iii) be clonal, amplified directly from tissue; and (iv) be sampled from the brain, or sampled from a patient for which the HBSD already contains brain sequences. We identified sequences for inclusion both by searching the public sequence databases--Genbank and the LANL HIV sequence database--and by identifying publications that sequenced HIV from the brain. In several cases, we communicated directly with study authors to encourage deposition of sequences that had not been previously submitted to Genbank. Additionally, BLAST alignment was used to screen for possible contamination with commonly used lab strains (i.e., ADA, HXB2, JR-CSF, NL4-3, SF2, BaL, IIIB, MN, SF162, and JR-FL)

### Annotation Structure

The HIV Brain Sequence Database contains three categories of annotations: publication references, patient and sampling information, and sequence properties (Table 3). The publication annotations include bibliographic information identifying the study that generated the sequences. Patient sampling annotations contain information describing the individual patients, as well as clinical information at the time of sampling. This information was obtained by manual curation of the original publications and in some cases direct communications with the study authors. In cases where multiple studies examined tissue samples from the same patient, the resulting sequences are linked to the same patient code to increase statistical power. Sample timepoint annotations describe the patient's clinical health status, neurocognitive, neuropathological status, CD4 counts, viral load, and anti-retroviral treatment history at the time of sampling. Clone and sequence annotations describe the individual sequences, the tissue from which they were cloned, and the method of PCR amplification and cloning. This includes the sequence start and end locations numbered based on alignment to the HXB2 reference genome, and tissue source coded using terms from a formal anatomical ontology. Alignment to HXB2 was performed using the HIV Sequence Locator tool located at the LANL HIV Sequence Database (http://hiv.lanl.gov). Currently, amplification and cloning methods included in the database are: bulk PCR then cloning (1736 sequences) and limiting-dilution PCR then cloning (781 sequences). As new sequencing projects are completed, we hope to expand the database to include significant numbers of sequences cloned via single genome amplification.

**Table 3:** Annotation categories

Patient	Column Definition
Patient code	patient code
Sex	gender
Risk factor	HIV risk factor
Tissue bank	tissue bank distributing samples
Patient year of death	patient year of death

**Sampling timepoint**	

Sampling geo-region	patient geo-region at time of sampling
Sampling country	patient country at time of sampling
Sampling city	patient city at time of sampling
Patient age	patient age at sampling
Health status	patient health status at sampling
Subtype	predominant subtype at time of sampling
Drug naïve (ART)	has patient had ART
Antiretroviral treatment (ART)	patient ART history
Viral load plasma (copies/mL)	plasma viral load
Viral load brain (copies/million cells)	brain viral load
Viral load lymphoid (copies/million cells)	lymphoid viral load
CD4 count (cells/uL)	CD4 count
Neurocognitive diagnosis	neurocognitive diagnosis
Neuropathological diagnosis	neuropathological diagnosis
Giant cells	were giant cells present in the brain

**Sequence**	

Genbank accession	Genbank accession number
GI	Genbank GI number
PubMed ID	Pubmed ID for original publicaiton
Sequence length	sequence length
Clone name	publication assigned clone name
Cloning strategy	methods of genome amplification and cloning
Sample tissue class	global tissue class (Brain, Blood & Lymphoid, etc...)
Sample tissue name	tissue source
Sample tissue FMA code	tissue FMA code
Nucleic acid type	was proviral DNA or viral RNA sequenced
Start and end coordinates	sequence start and end referenced to HXB2
Sequence	viral sequence

### Annotation of Tissue Type

Annotation of tissue source presented several challenges. First, the granularity of tissue annotation varied by publication--we encountered tissue type annotations as general as "Brain" and as specific as "White matter of occipital lobe". However, within the HBSD a search for a more general tissue type, such as cerebrum should also return sequences from sub-parts of the cerebrum, such as caudate nucleus and putamen. Second, publications utilize non-standard tissue names that are human-readable but difficult to parse in a database search. To address these challenges, we utilized a formal anatomical ontology, the Foundational Model of Anatomy (FMA) to code tissue source [14]. The FMA defines terms for approximately 75,000 human anatomical structures, ranging in scale from biological macromolecules to whole organ systems. These terms are linked by ontological relationships defining subpart relationships, allowing the calculation of transitive closure within the database. In addition, we assigned sequences into one of four classes: (i) Brain; (ii) Meninges, choroid plexus, and CSF; (iii) Blood and lymphoid; and (iv) Other. Meninges, choroid plexus, and CSF were grouped separately from Brain because phylogenetic evidence suggests that the CSF represents an intermediate compartment, containing virus from both the brain and periphery [8]. "Other" includes organs such as lung, liver, stomach and prostate, bone marrow, and fluid samples such as lung epithelial lining fluid.

### Annotation of Neurocognitive and Neuropathological Diagnosis

Neurocognitive and neuropathological status were classified for each patient at the sampling timepoint, usually perimortem (Table 4). Neuropathological and neurocognitive disorders can be due either to virus replicating in the brain or to non-HIV related causes such as toxoplasmosis, CMV encephalitis, or CNS lymphoma. Neuropathological status was coded as HIV encephalitis (HIVE) of varying severity, lymphocytic perivascular cuffing, or "Other", specifying the predominant non-HIV neurological pathology. Neurocognitive diagnosis was annotated using the nomenclature consensus published in Antinori et al, 2007 [4]. We further classified the HAD diagnosis into mild, moderate, and severe to capture information included in the publication as mild, moderate, or severe (most commonly) or MSK scores (rarely). Additionally, there were several unique cases that fell outside the AAN or HNRC criteria, but which we felt were important to annotate within the database. Diagnosis for patient 196 stated: "insufficient information for patient 196 for the diagnosis of HAD, though there was evidence for neuropsychiatric disease."[15]. Given that we lacked the further information to meet the strict criteria for an ANI or MND diagnosis, we chose the more general NPI: unknown defined in Woods et al. 2004 [16]. Diagnoses for patients 1 through 6 stated, "Clinical material was obtained from six HIV-1 infected patients with significant neurological signs and symptoms requiring image-guided stereotactic brain biopsy for definitive diagnosis. ... Neurological signs and symptoms were consistent with the onset of global neurological dysfunction, with clinical evidence supporting acute rather than chronic HIV-1-associated neurological disease."[17]. As an acute diagnosis, this does not fit the criteria for HAD, so it was annotated in the database as acute HIV encephalopathy [17].

**Table 4:** Neurocognitive and neuropathological annotations in the database

Neurocognitive Diagnosis	Number of Patients	Number of Sequences
None	42	739
Acute HIV encephalopathy	6	209
HAD: mild	7	46
HAD: moderate	4	8
HAD: severe	11	424
HAD: severity not specified	19	810
NPI-unknown	1	10
No diagnosis	7	271

**Neuropathological Diagnosis**		

None	37	369
HIVE: mild	5	276
HIVE: moderate	3	100
HIVE: severe	6	117
HIVE: severity not specified	17	938
Lymphocytic perivascular cuffing	1	31
Other: cerebral atrophy	1	39
Other: CMV encephalitis	1	5
Other: CNS lymphoma	7	242
Other: necrotizing encephalitis, not HIV-related	1	23
Other: progressive multifocal leukoencephalopathy	2	36
Other: toxoplasmosis	3	83
Other: widespread atherosclerosis	1	87
No diagnosis	12	171

An annotation of "none" indicates a diagnosis of no impairment or neuropathology, whereas "no diagnosis" indicates that clinical annotation information was not available.

### Design and Implementation

**Figure 2:**
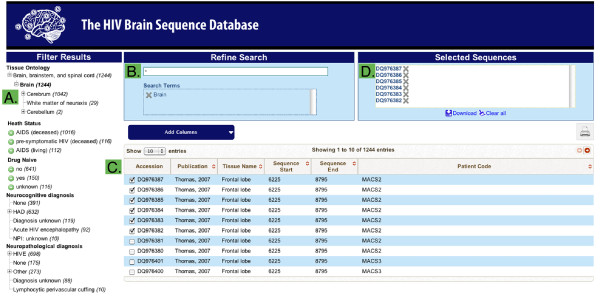
**Search interface of the HBSD**. A. Database facets for filtering results. All possible values for each category are presented, along with a count of the number of sequences for each value. Clicking on a value adds it to the search box (B), filters the results list (C), and updates the facet list and sequence counts (A). B. Universal search box and search term list. Performs a global search across categories, for example, a search for "right" returns sequences from both "Right frontal lobe" and "Right lung". Upon searching, the facet list (A) and results (C) are updated. All searches and faceting terms applied are placed in the Search Terms box and can be removed individually by clicking the "X" next to a term. C. Results list. Displays the current list of sequences matching the filters within the Search Terms box (B). Columns can be added or removed through the Add Columns button. Clicking the checkbox by a sequence adds it to the Selected Sequences box (D). D. Selected Sequences and Downloads. Clicking the download button presents options to download: (i) Current Results--all sequences matching the search terms, (ii) Current Selection--all selected sequences in box D, (iii) Entire Collection--the entire HBSD. Downloads consist of a zip file containing a FASTA formatted file of all sequences, named by Genbank accession number, and a tab-separated file of all selected annotation columns, ready for import to Excel.

The HBSD structure is sequence-centric and uses NCBI GI and Genbank accession numbers as identifiers, simplifying correlations with other databases. The database exists in two forms. The master version is kept internally as a relational SQL database utilized for sequence management and curation. This is replicated to an external interface that uses the Apache Solr search platform to optimize for flexible search and data retrieval. The search interface (Figure 2) is based on a filtering paradigm; the user begins with the set of all sequences and narrows by applying filtering criteria to the sequence annotations. Filtering criteria are specified by two means. A faceted search interface presents all values for categorical annotations, such as tissue class or neurocognitive status. Clicking on a value adds it to the search criteria and filters for matching sequences. Additionally, a global search box allows direct entry of search terms. Multiple searches in the global search box sequentially add filtering criteria, allowing the construction of complex searches. Sequences are initially presented with a default set of annotations, however, users can select to add or remove columns from the set of all annotations available. The final filtered set of sequences and annotations can be downloaded for local analysis in tab-separated and FASTA formats.

### Visualization of the contents of the database

**Figure 3:**
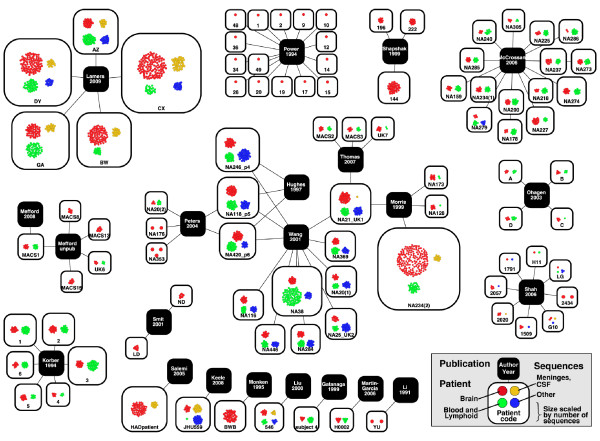
**Network representation of interconnections between publications, the patients they sequenced, and the number and tissue classes of sequences available for each patient**. The network was constructed using Cytoscape. Black nodes, containing the name of the first author, represent publications. Publication nodes are connected by edges to the patients they sequenced, represented by clear nodes with patient code printed at the bottom. In cases where multiple publications sequenced virus from the same patient, multiple publication nodes connect to a single patient node (patient NA118 in the upper right). Individual HIV sequences for each patient are represented by the colored dots within patient nodes: Brain-red, Meninges, choroid plexus, and CSF-yellow, Blood and Lymphoid-green, Other-blue. The total number of sequences for each patient scales the size of the patient node.

To better understand the highly complex network of publications, patients, and sequences, we used Cytoscape to visualize the connections between patients and the publications that sequenced virus from those patients (Figure 3). This network visualization demonstrates that, while most publications examine a unique set of patients, there is an emerging network of patients from the Edinburgh MRC HIV Brain and Tissue Bank (coded as NA#) that are shared among multiple publications. Additionally, Figure 3 illustrates the dramatic differences in sequencing depth between patients, and in number of patients between studies.

**Figure 4:**
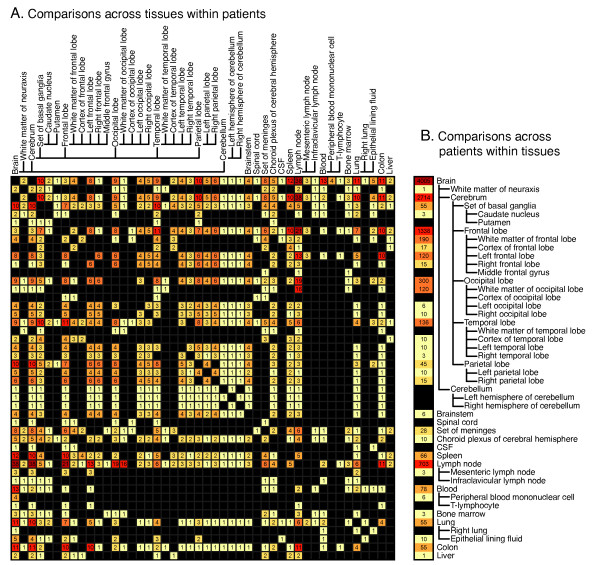
**Heatmap representation and counts of all possible comparisons between sets of overlapping sequences within the database**. Counts of possible comparisons were generated using 2 custom Perl scripts and SQL statements, then visualized as a heatmap using R. A. Number of patients for which within-patient comparisons across tissue-types can be made. For pairs of tissues from the X and Y-axis, numbers indicate the number of patients for which overlapping sequences from both tissues are available. For example, there are 11 patients with overlapping sequences from both Frontal lobe and Temporal lobe. B. Number of possible pair-wise comparisons across patients within each tissue type. For each tissue on the Y-axis, numbers indicate the count of possible pair-wise comparisons between patients. For example, there are 2 patients with overlapping sequences from White matter of neuroaxis, giving 1 possible comparison, and 4 patients with overlapping sequence from Left occipital lobe, giving 6 possible pair-wise comparisons. Tissue definitions are ontologically inclusive, i.e. Frontal lobe also includes White matter of frontal lobe, Cortex of frontal lobe, etc. Cells are colored as a heat map accentuating high values, and range from light yellow (low values) to dark red (high values). Black indicates no comparisons possible.

Many experimental designs examining compartmentalization or tissue specific effects depend on overlap in the viral regions sequenced and matched tissue source. In order to quantify the power of the database to make these comparisons, we visualized the total number of across-tissue and within-tissue comparisons possible with the current database content (Figure 4). Panel A visualizes, for each tissue pair, how many patients contain overlapping sequences. Each comparison is ontologically inclusive--for example entries under Frontal lobe also consider sequences from White matter of frontal lobe, Cortex of frontal lobe, etcetera. This visualization reveals structures within the dataset useful for experimental design. For example, while a large number of patients contain overlapping sequences from lymph node and another tissue, in 8, 11, and 7 patients, respectively, it is possible to compare frontal lobe to occipital, temporal, or parietal lobes. Figure 4B is a complementary visualization counting the number of pairwise patient to patient comparisons possible within each tissue type. This illustrates, for example, that while many patients have overlapping sequences from the cerebrum, frontal lobe is a particularly well-represented tissue. Conversely, though the database contains sequences from the cerebellum, there are no across patient comparisons that can be made. The numbers in both A and B of Figure 4 do not represent simple sums or permutations, because each considers sequence overlap. If hypothetical patients A, B, and C contained full-length *env*, V3 region, and V5 region sequences, respectively, then only 2 pair-wise comparisons would be possible (A to B and A to C), not the 3 given by a simple permutation.

## Discussion

The HBSD is a public database designed to facilitate the assembly of a large meta-dataset of HIV *env *sequences that will be invaluable to investigations into the different patterns of viral evolution in the brain and other tissue reservoirs, and the relationship of these findings to each other and to clinical consequences of HIV infection, particularly development of HAND. The database contains 2517 *env *sequences cloned from 90 patients and 44 tissues sources. 1272 of these sequences are brain-derived; the remaining 1245 are derived from blood, lymph node, spleen, bone marrow, colon, lung, and other non-brain tissues. The majority of these sequences are from the V3 region (45%) or near full-length gp120 region (31%), with the remainder being near full-length *env *(9%), V4-V5 region (6%), V1-V2 region (4%) and others (5%) (Figure 1). The HBSD is unique compared to other sequence databases, such as the LANL HIV Sequence Database or Genbank, because of its specific focus on HIV in the brain, its stringent inclusion of only clonal sequences from patients with brain sequences, and its rigorous curation with detailed clinical, patient, and HAND annotations.

An HIV *env *meta-dataset annotated with detailed clinical information will allow studies that previously have not been feasible. Combining datasets to increase the number of sequences and tissue-types increases the statistical power available. This increased statistical power can be used to examine questions such as the genetic variations within *env *important for macrophage tropism, which is the primary requirement for HIV replication in the brain, and nucleotide positions within *env *under positive genetic selection during HIV replication in the CNS. Annotation of neurocognitive status, neuropathological status, and AIDS progression will facilitate correlation of viral genotype to clinical phenotypes, and may help to reveal how viral genotypes affect the development of HAND.

During the assembly and annotation of the HBSD, we encountered a number of challenges. Non-uniform tissue coding made consistent database annotation difficult. To overcome this obstacle, we utilized the FMA anatomical ontology to convert various tissue source descriptions into a set of defined terms with ontological linkages. We encountered several instances of ambiguous patient coding. Because tissue samples are shared within laboratories, and tissue banks distribute samples from the same patient to multiple laboratories, viruses from one patient may be sequenced in multiple publications. By examining patient annotation data and corresponding with study authors, we identified 3 patients that were coded differently by multiple studies (NA118_p5, NA420_p6 and NA21_UK1) and 2 cases of separate patients that were coded identically by different studies (NA20 and NA234). Combining sequences from multiple publications and grouping by patient can increase the diversity of tissue types and the depth of sequencing available, while carefully tracking patient coding can avoid incorrect grouping of non-identical patients. Many publications included in the HBSD contain duplicate sequences cloned from the same tissue sample. These duplicate sequences could result either from PCR resampling in studies utilizing bulk PCR before cloning, or could represent valid cloning of copies of a majority viral variant. Fifteen publications utilized bulk PCR then cloning, 5 utilized limiting dilution then cloning, and 2 used both approaches, based on patient. The database contains 490 repeated sequences in 161 groups. However, 217 of these repeated sequences were obtained by limiting dilution PCR and therefore are unlikely to represent PCR resampling. Comparison of the distribution of the percentage of duplicated sequences between bulk PCR and limiting dilution demonstrated that studies utilizing bulk PCR then cloning did not show a higher rate of sequence duplication than those utilizing limiting dilution (data not shown). Thus duplicated sequences in the database likely represent appropriate cloning of majority viral variants.

The HBSD includes several unique datasets, which, though previously available in the public domain, are now collected in a standardized annotation format for meta-analysis. 15 patients included in McCrossan, 2006 [18] are pre-symptomatic, having died from HIV-unrelated causes [alcohol/drug overdose (n = 11), cirrhosis (n = 2), suicide (n = 1), and bronchopneumonia (n = 1)]. During late-stage AIDS, declining CD4 counts lead to immune deficiency and reduced selection pressure, allowing viral population expansion that may alter the distribution of sequence variants. Based on treatment history and year of death, the majority of patients in the HBSD died prior to the HAART era. 49 out of 90 patients have annotations for antiretroviral treatment history. Of these 49 patients, 19 are drug naïve and 30 received antiretroviral drugs. The majority of antiretroviral treated patients were on pre-HAART regimens, and 9 received only AZT. Different ART drugs have differing CNS penetration, affecting selection pressures on virus replicating in the brain [6]. Additionally, the majority of neurocognitive diagnoses occurred before the 2007 HNRC consensus document [4] that defined criteria for asymptomatic neurocognitive impairment (ANI). Future improvement of the quality and relevance of the database to the current epidemic requires generating more sequences sampled from the brains of pre-symptomatic patients at earlier stages of disease and HAART-treated patients.

Our laboratory will continue to maintain the HBSD as new sequences are deposited in the public domain. We expect the HBSD to expand in several ways. New deep sequencing projects will increase the number of sequences and expand the diversity of patients, sampling a wider spectrum of stages of disease and HAART treatment regimens. Curation of patient coding may allow us to identify longitudinal sets of sequences sampled from the periphery, which can be paired with brain sequences sampled from the same patient at autopsy. Finally, we chose to focus on *env *for the initial database release because it plays a key role in brain infection and provides a tractable scope for development of a highly curated database. As we consider further database additions, we will continue to weigh the benefits of inclusion against the resources required to maintain our high standards of database curation. *Tat *and *nef *are two logical next steps, as these genes influence brain infection and development of neurocognitive disorders. Drug resistance mutations in *pol *and RT would also be a useful addition that will be considered in the future.

## Conclusions

The HBSD is a unique resource for the research community investigating unique genetic and biological characteristics of HIV in the brain. Though nearly all the sequences and annotations included were previously available in the public domain, the data did not exist in a well-annotated and accessible format and its assembly and curation represented a significant hurdle. The HBSD will be an invaluable resource for studying the viral genetics of HIV evolution within the brain and other tissue reservoirs, and the relationship of these findings to each other and to the development of HIV-associated neurocognitive disorders.

## Competing interests

The authors declare that they have no competing interests.

## Authors' contributions

AH designed the sequence database, assembled and curated sequences, performed all bioinformatic analysis, and drafted the manuscript. MM assembled and curated sequences and clinical data. NO designed and implemented the database interface. DG conceived of the study, participated in its design and coordination, and helped to draft the manuscript. All authors read and approved the final manuscript.
